# Adjusting to university: Perceptions of first-year health professions students

**DOI:** 10.1371/journal.pone.0251634

**Published:** 2021-05-25

**Authors:** Bunmi S. Malau-Aduli, Mary D. Adu, Faith Alele, Karina Jones, Aaron Drovandi, Martina Mylrea, Kornelija Sfera, Simone Ross, Ernest Jennings

**Affiliations:** College of Medicine and Dentistry, James Cook University, Townsville, Australia; Universidad Nacional de Educacion a Distancia, SPAIN

## Abstract

**Background:**

The transition experience into university can be challenging for health profession students as they are required to rapidly learn diverse and adaptable problem solving skills and advanced reflective thinking processes which are necessary to address complex patient-care problems, particularly in the face of uncertainty within a dynamic and rapidly evolving learning environment.

**Methods:**

A mixed-methods study was conducted to identify factors influencing this transition for first-year medical, dental, and pharmacy students at a regional Australian university. The Student Adaption to College Questionnaire (SACQ) examined participants’ levels of adjustment to university, while Schlossberg’s 4 S transition model was utilised in a framework analysis of the focus group and interview responses.

**Results:**

Complete survey responses were obtained from 198 students, 17 of whom also participated in focus group discussions or interviews. Mean adjustment ratings obtained from the SACQ responses were academic (6.09 ± 1.3) personal-emotional (5.53 ± 1.55), social (6.30 ± 1.38), and institutional attachment (6.96 ± 1.6). These results indicate that the personal and emotional aspects of this transition were more challenging for the students. Analysis of the qualitative data revealed that generally, for these highly motivated health-professions students, dropping out of university was not an option and this had a positive influence on their ability to adjust to their new learning environment. Nonetheless, the transition involved role change; school-leavers were excited about their newly found independence, while for mature-aged students, returning to university allowed them to pursue their lifelong dreams. Adjustment was more challenging for international, mature-aged and female students, with personal and social factors influencing the transition for each of these demographic groups.

**Conclusions:**

To facilitate smooth transition into university, tertiary education institutions must consider tailored on-going support strategies that promote social interaction among students with varied backgrounds and personal characteristics.

## Background

Adaptation into the university environment creates significant stress and uncertainty for students, including those enrolling immediately after high school, as well as mature-aged and international students [1, 2]. This stress and uncertainty stems from a combination of having unrealistic expectations of the university experience, and being faced with different challenges in relation to approaches to teaching, learning, assessment, financial and social adjustment issues [3, 4].

Effective integration into university is a significant predictor of institutional reputation, student retention, and academic success among first-year students [5] and this has made transition into university an important and growing field of research in higher education [6]. There has been a wide range of predictors identified (with varying degrees of relevance) which impact on the transition of students into the university environment [5, 6]. Apart from pre-existing expectations and previous academic achievement or experiences, other components of university life which may affect retention and student outcomes include the orientation to the institution, social demands, motivation and other personality traits, concurrent responsibilities and support systems [6–8]. In addition to enhancing existing supportive measures, the early detection of potential adjustment problems during this key transitional stage could reveal areas requiring additional student support [1]. Understanding the needs, priorities, and previous support-related experiences of the student population leads to improved targeting of cost-effective interventions and support services to enable successful transition into university and completion of studies [1, 9, 10].

Four major adjustment domains are reported to be associated with first year students’ transition into university experiences–these include academic, social, personal-emotional and goal/institutional adjustments [11, 12]. Academic adjustment describes how well students are able to cope with educational demands, such as motivation to complete academic requirements, academic effort, performance and satisfaction with the environment. Social adjustment refers to the interpersonal-societal demands intrinsic in adjustment to university, which include students’ participation in social activities and experiencing fulfilment with various aspects of their university experience. Personal-emotional adjustment denotes the psychological and physical feelings of students. Finally, the goal/institutional adjustment refers to students’ satisfaction with university in general, and the institution of attendance. These multifaceted categories are inherent determinants of how students are able to cope with the new demands and stress of university life [11, 12].

Previous research investigating factors affecting the transition into university have been mostly limited to single degree pathways, with medicine dominating the current literature within the wider health professions field and some of the issues identified include psychological distress, emotional exhaustion and financial pressure [3, 13–16]. This reduces the applicability of the findings across diverse health profession disciplines, as students in different disciplines may have similar or different expectations associated with their chosen career pathway and experiences in their progression through that pathway. Smooth transitioning into university is particularly important for health profession students, because they are required to have or rapidly learn diverse and adaptable problem solving skills and advanced reflective thinking processes which are necessary to address complex patient-care problems [17, 18]. Additionally, health profession students must possess good coping strategies, study skills, motivation and resilience, particularly in the face of uncertainty within a dynamic and rapidly evolving learning environment [19–21]. Consequences of inadequate transition in the areas of academic, emotional, social and institutional adjustment include elevated levels of stress and anxiety, poor academic performance, inability to integrate socially as well as dissatisfaction with the learning environment and these may result in a decision to drop-out of university [7, 22, 23].

Therefore, this paper aims to identify the factors that influence first year medical, dental, and pharmacy students’ transition experiences at a regional Australian university. It explores a range of factors that influence their experiences in the areas of: academic adjustment, social adjustment, personal-emotional adjustments, and institutional attachment. The paper also proffers recommendations on facilitating smooth transitioning into university and improving students’ learning experiences.

### Organisational context

James Cook University’s (JCU) College of Medicine and Dentistry (CMD) offers undergraduate training in medicine, dentistry and pharmacy. JCU is one of the 37 public universities in Australia and student admission procedure into these CMD degrees involves a combination of national tertiary entrance examination, written application and a semi-structured interview (for medicine only). All three degree pathways are full-time, internal delivery (on-campus only) programs with pre-clinical training across the fields of anatomy, physiology, chemistry, biochemistry, healthcare systems, and introductory materials to the relevant discipline. This first year introduces students to short observational placements in the three disciplines, which become more interactive as the students progress into the clinical years of their respective degrees. These degrees also have a focus on tropical, rural, and remote medicine, which relate to JCU’s physical placement in North-Eastern Australia and purpose in servicing the health needs of regional, rural, remote and Australian Aboriginal and Torres Strait Islander communities.

The CMD recognises that transition to university can be stressful and offers support to its students in the form of academic advisors, home group and health professional self-care (HPSC) programs. Academic advisors are faculty with whom academically ‘at-risk’ students can discuss issues and who refer students to counselling, medical, crisis and academic support services. The HPSC program has been provided to first year students for the past three years. It consists of eight sessions throughout the year aimed at helping students to recognise and manage stress, and develop coping mechanisms; as well as teaching stress-relieving techniques to improve student wellbeing, and reduce study/ exam-related stress [24]. Home groups are currently offered to students studying medicine and dentistry only. The program provides the students with social, professional and academic support in small groups of 8–10 individuals. Each home group is facilitated by a staff member and/or a senior student. The facilitators aim to help the new students assimilate into university life via social and regional orientation to their fellow students, their discipline and the university campuses. They also orient students to academic activities in a supportive educational environment and cover a range of topics including study skills, critical thinking, theoretical case studies and practice examinations.

## Methods

This research employed an explanatory sequential mixed-methods design [25], which included the collection and analysis of both quantitative (survey) and qualitative data (focus groups or interviews) from first-year undergraduate medical, dental and pharmacy students at JCU. The qualitative data was collected to provide a comprehensive understanding of the quantitative results, which were integrated in the data interpretative phase.

### Participant recruitment

All 2019 first-year students enrolled in the medical (n = 206), dental (n = 101) and pharmacy (n = 50) programs were invited in September—October 2019, to participate in survey and interview or focus group discussions (FGDs). Collation of data in the second semester of study allowed students have deeper insights into their transition experiences. Students were recruited through an announcement during one of their scheduled class times one week before the scheduled survey date. Students were assured of no adverse academic repercussions for non-participation, with staff not associated with the teaching of students involved in all data collection processes. As an incentive, participants from each discipline were entered into a separate draw to win one of three available $50AUD gift cards. Ethics approval for the study was obtained from the JCU Human Research Ethics Committee and participation was voluntary.

### Surveys

The Student Adaptation to College Questionnaire (SACQ) [11, 12] is a validated 67-item self-reported survey that was used across all disciplines in this study. The survey was used with permission from Western Psychological Services (Torrance, California). The survey items gathered data on students’ self-perceived adjustment to the university environment, including their academic (24 items), social (20 items), emotional (15 items), and goal commitment /institutional adjustment (8 items). Individual items within each of these types of adjustment are grouped into clusters (e.g. motivation, performance, nostalgia, psychological and physical adjustment). The SACQ contains statements on a 9-point Likert-scale, rated from 1 (does not apply to me at all) to 9 (applies very closely to me), where higher scores signify better adaptation. Surveys were administered in class between August and September 2019. Collected demographic information included age, gender, rurality, origin, previous degree, health professionals in the family, first in the family to attend university. In addition to the SACQ items, participants responded to three open-ended questions which were related to: 1) factors that have been beneficial in the transition, 2) the most challenging aspects of transitioning into university, and 3) additional support measures that could be provided to students during the transition. The last question in the survey was used to identify participants who were interested in participating in the qualitative interviews.

### Focus group discussions/interviews

FGDs and interviews were conducted in October 2019 to obtain in-depth understanding of participants’ transition experiences. Semi-structured open-ended questions were generated based on survey findings and existing literature, and utilised to facilitate discussions. The FGDs/interviews were conducted by BSMA who is an experienced qualitative researcher. MDA and KJ served as observers to ensure appropriate data acquisition. The aim of the study was reiterated and verbal consent was obtained from the participants before commencing the FGDs/interviews. The FGDs were conducted in secure private rooms at JCU. For the four interviews that were conducted via telephone, the participants were asked whether they were in a convenient location before the interviewer proceeded with the interview. The discussions were audio-recorded, lasted up to 60 minutes, and took place in informal group settings, with emphasis on the importance of honesty and confidentiality. Only staff not associated with the teaching of the students (BSMA, MDA and KJ) were involved in the focus groups/interviews. No relationships between the interviewer and participants were established prior to study commencement. The FGDs and interviews continued until data saturation was achieved, that is the participants’ responses were no longer revealing new information [26].

### Ethics approval and consent to participate

The James Cook University Human Ethics Review Committee granted ethics approval for the study (H7853). Written informed consent was obtained from study participants following invitation which was accompanied by an information sheet.

### Data analysis

Survey data were analysed using SPSS v25 (IBM Corp. Armonk, NY, USA). As suggested by the survey developers [27], completed surveys with one or two missing responses were prorated using an average of other responses in the subscale, while those with more than two missing responses to items in each subscale were removed from the analysis. The average score across each subscale was calculated to give an index of adjustment within each domain. Consistent with previous research [12], Cronbach alpha for the SACQ domains ranged from 0.80 to 0.84. Pearson’s correlation coefficients were used to establish the underlying correlations between the sub-domain questions. Age was collapsed into three groups (17–19, 20–25, 26 and older) for analysis. Using exploratory analysis, the effect of students’ socio-demographic characteristics on their adaptation scores were assessed. T-test was used for variables with two categories (gender, previous degree, health professionals in the family, first in the family to attend university) while ANOVA was used for variables with three or more categories (age group, rurality, origin).

**Fig 1:**
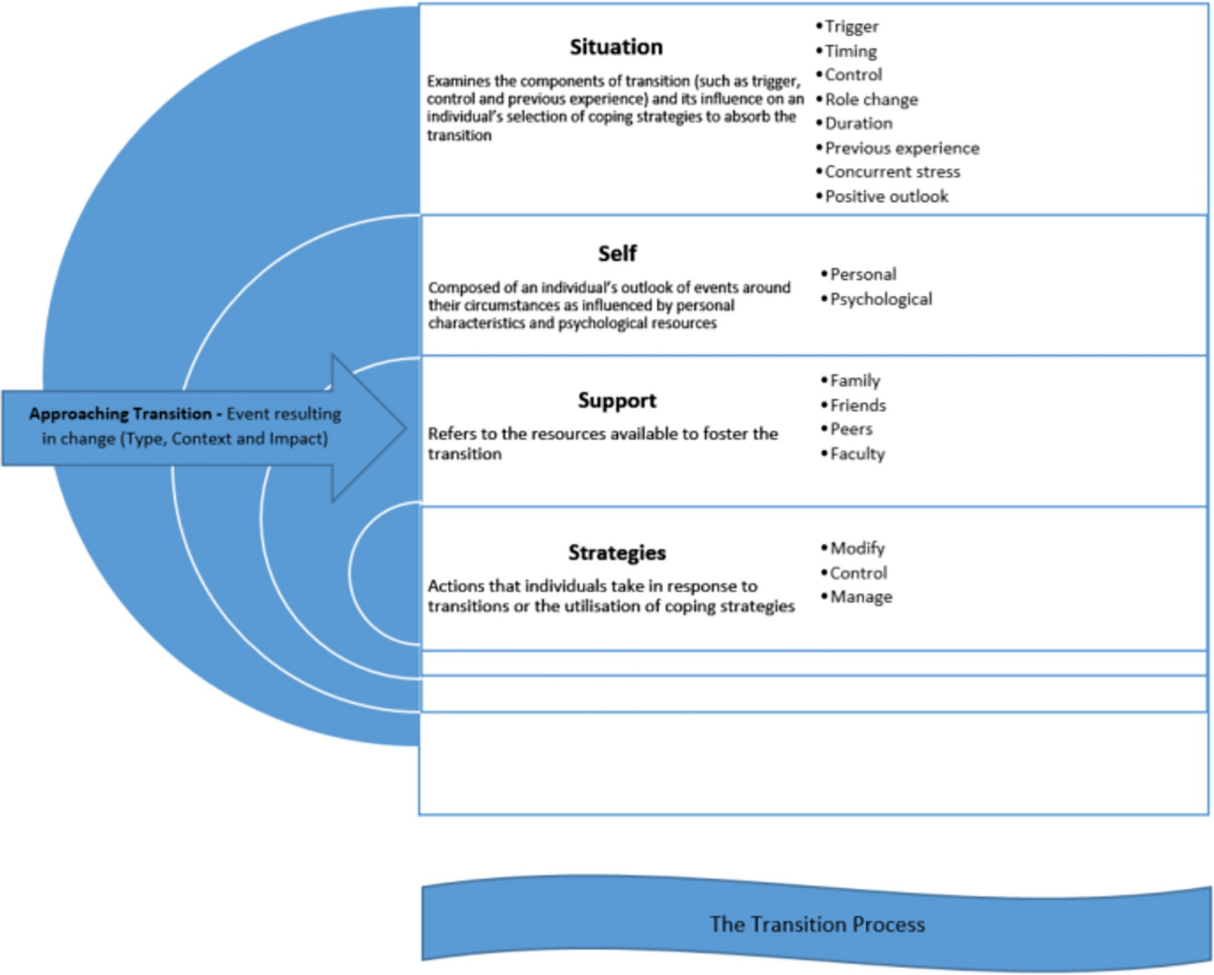
Schlossberg’s transition theory.

Qualitative data was stored and analysed in QSR NVivo 11. Open-ended survey responses and FGDs/interviews were transcribed and coded using a line-by-line open coding process and constant comparison process as advocated by Corbin and Strauss [26]. The transcripts were subjected to framework analysis by applying the concept driven theoretical 4 S transition model developed by Schlossberg et al. [28]. As shown in Fig 1, Schlossberg et al. [28], posit that *transition* experience and the resulting outcomes are influenced by many factors, which are grouped into four major categories: situation, self, support, and strategies (known as the 4 S). These four categories influence a person’s ability to cope with transition and also determine how different individuals react differently to the same type of transition. This theory has been applied to a range of transitional research involving adults, in relation to their into and throughout university experiences [29, 30]. In this study, the 4 S model was used to identify, map, and explain the qualitative findings, allowing in-depth understanding of the concept of transition into university from the perspective of first year health profession students. A deep understanding of the transition experience of this group of students viewed through the lens of Schlossberg et al.’s 4 S model may facilitate a smooth transition process for them. See Fig 1.

Emerging themes were identified and independently confirmed by three researchers (MDA, FA and BSMA) in order to enhance result credibility and trustworthiness. These researchers have experience in qualitative research methods. Identified themes are presented using illustrative quotes from the open-ended survey responses, FGDs and interviews. The consolidated criteria for reporting qualitative research (COREQ) checklist was developed to promote explicit and comprehensive reporting of the interviews and FGDs (See S1 Checklist).

## Results

### Participants’ characteristics

Survey responses were received from 57% of invited participants (203/357 students), 198 of which were complete. Response rates within the medicine, dentistry and pharmacy disciplines were 46%, 62% and 78% respectively. Demographic characteristics across all disciplines were similar and analysed collectively. The mean participant age was 19.87 ± 4.41 (range 17–48 years) and 61% were female, which is consistent with the CMD demographics. A larger proportion of the participants were medical students (48%) and enrolled as domestic students (82%). Over half of the respondents (52%) indicated that they have family members who are health professionals; 48% were from regional areas; 74% have no previous health professions education experience and 25% were first in their family to attend university. Please see Table 1 for details. A total of 56 (28%) respondents expressed interest to participate in the FGD or interview. However, only 17 (30.4%) of them participated in one of the four FGDs or four interviews (Females = 14, Males = 3).

**Table 1:** Demographic information of participants (N = 197).

	Frequency	Percentage
**Discipline**		
Dentistry	63	32.0
Medicine	95	48.2
Pharmacy	39	19.8
**Gender**		
Male	74	37.6
Female	121	61.4
Missing	2	1.0
**Enrolment status**		
Domestic	161	81.7
International	34	17.3
Missing	2	1.0
**Age**		
17–19	144	73.1
20–25	35	17.8
26 and above	12	6.1
Missing	6	3.0
**Health Professionals in Family**		
Yes	103	52.3
No	92	46.7
Missing	2	1.0
**Previous Degree**		
Yes	49	24.9
No	146	74.1
Missing	2	1.0
**Prior Health Experience**		
Yes	35	17.8
No	159	80.7
Missing	3	1.5
**First in the family to attend University**		
Yes	29	14.7
No	168	85.3
**Rurality**		
Major city	45	22.8
Regional Centre	95	48.2
Rural/Remote Communities	53	26.9
Missing	4	2.0

### Survey results

#### Adaptation to University

Cronbach’s alpha indices were 0.84, 0.84, 0.81 and 0.80 for the academic, social, emotional and institutional domains respectively. The overall mean score for academic adjustment was 6.09 ± 1.3, with students scoring highest in the motivation subscale (6.82 ± 1.72) and lowest in the performance subscale (4.96 ± 1.8). With regards to social adjustment, participants’ mean score was 6.30 ± 1.38, with best adjustment to the environment (6.65 ± 1.7). Emotional adjustment scores were relatively similar across the two subscales of psychological (5.52 ±1.6) and physical adjustments (5.54 ±1.7) with a mean score of 5.53 ± 1.55. Participants scored higher in the domain of adjustment to achieving their goals (7.6 ± 2.2) compared to their adjustment to the university (6.33 ± 1.22). Please see Table 2 for details.

**Table 2:** Overall mean scores with each domain of adaptation to university.

			Correlation											
Main Domain	Sub- Domain	Mean + SD	1	2	3	4	5	6	7	8	9	10	11	12
**Academic Adjustment**	1] Motivation	6.82±1.76												
	2] Application	5.96±1.80	.587^**^											
	3] Performance	4.96±1.80	.254^**^	.564^**^										
	4] Academic Environment	6.60±1.61	.682^**^	.467^**^	.366^**^									
	**Total**	6.09±1.30												
**Social Adjustment**	5] General	6.34±1.58	.618^**^	.327^**^	.184^**^	.577^**^								
	6] Other People	6.29±1.38	.613^**^	.393^**^	.218^**^	.594^**^	.717^**^							
	7] Nostalgia	5.92±2.02	.371^**^	.346^**^	.424^**^	.438^**^	.483^**^	.541^**^						
	8] Social Environment	6.65±1.70	.665^**^	.346^**^	.229^**^	.637^**^	.696^**^	.654^**^	.471^**^					
	**Total**	6.30±1.38												
**Emotional Adjustment**	9] Psychological	5.52±1.60	.291^**^	.421^**^	.680^**^	.393^**^	.284^**^	.307^**^	.595^**^	.335^**^				
	10] Physical	5.54±1.70	.389^**^	.485^**^	.602^**^	.471^**^	.375^**^	.362^**^	.525^**^	.399^**^	.757^**^			
	**Total**	5.53±1.55												
**Goal Commitment/Institutional Attachment**	11] General Attachment	7.60±2.22	.820^**^	.430^**^	.218^**^	.622^**^	.594^**^	.569^**^	.395^**^	.687^**^	.354^**^	.384^**^		
	12] This University	6.33±1.22	.731^**^	.478^**^	.320^**^	.735^**^	.784^**^	.825^**^	.656^**^	.778^**^	.446^**^	.479^**^	.696^**^	
	**Total**	6.96±1.60												

**Correlation is significant at the 0.01 level (2 tailed)

#### Exploratory analysis of demographic variables

Male participants had higher scores in emotional adjustment when compared to their female counterparts, t (197) = 2.35, *p* = 0.02, eta squared 0.01. Additionally, having a health professional in the family significantly improved social adjustment, t (197) = 2.17, *p* = 0.03, eta squared 0.02. There were significant age differences in social adjustment, with students aged 26 and above adjusting better F (2, 194) = 3.25, *p* = 0.04, eta squared 0.03. Mean scores for social adjustment among those aged 26 years and above (7.05 ± 1.17) was significantly higher than for those aged 20–25 years (5.90 ± 1.650) and 17–19 years (6.34 ± 1.31). Furthermore, having prior health education experience significantly influenced academic adjustment (t (194) = 2.93, *p* = 0.004); social adjustment (t (194) = 3.24, *p* = 0.002) and institutional adjustment (t (194) = 2.74, *p* = 0.000). See S1 Table for details. There were statistically significant (p < 0.01) positive correlations (ranging from .18 to .82) between sub-scale domains. See Table 2 for details.

### Qualitative results

#### The 4 S model of transition: Situation, self, support and strategies

Qualitative study findings are presented in an interpretative way and underpinned by Schlossberg et al.’s [28] theoretical transition model. Illustrative quotes are reported verbatim, and each one is depicted using participant gender (M/F) and an assigned number, response method—FGD or Interview (I) and discipline of study—Medicine (Med), Dentistry (Dent) and Pharmacy (Pharm). (Example—F4, FGD, Med).

#### Situation

*Trigger*: *What set off the decision to study at JCU*?. Students in all three disciplines identified the rural focus, tropical medicine and research mission of JCU as key contributing factors which triggered their interest to pursue their degree at this institution. This decision was further boosted by the alignment of the JCU mission with their future career goals.

*‘‘I fell in love with what the university had to offer*. *It focusses so much on tropical medicine and rural remote health*. *That’s something relevant to what I want to do in the future as a doctor*.*”* [F3, I, Med]

For school-leavers, the decision to go to university was fostered by recommendations from recruitment agencies, health professionals and family.

*‘‘I always knew I wanted to go into Dentistry because my uncle’s a dentist*. *So*, *I grew up watching him and observing the practice and understanding the field*.*”* [F1, FGD, Dent]

While for mature-aged students, returning to university to study was triggered by changed family circumstances that allowed them to pursue and fulfil their lifelong dreams. However, the factors that influenced the choice of study discipline varied among the age groups.

*‘‘Well*, *working with kids*, *that was my father’s dream for me*, *and that was the only like financial opportunity that we—our family could afford*, *which was free*, *that’s why [Laughter]…we could afford it*. *I always—followed what my parents were asking me*. *But at a certain point*, *when I did have my own kids*, *I decided that that’s the time*. *When they all went to school*, *I decided that’s my time to go to school and do something for me*, *finally…”* [F1, I, Dent];

*‘‘I gave up a lot to come here*, *and I think that adds pressure because I sold the home and left a job where I was very comfortable*. *I was making a good income*. *I was very established in my community*. *My children are overseas*, *and they’re all over-18*, *they’ve all left home*.*”* [F3, FGD, Pharm]

*Timing*. The school-leavers were deemed to be “on time” as coming to university is the most appropriate next phase of their lives and this allowed them to have relatively easier transitioning into their degree programs. However, the same cannot be said of the mature-aged students. Although the transition was anticipated, timing made it more challenging for this group of students. These students acknowledged that coming to study at an older age was associated with a lot of challenges including struggling with the academic schedule, use of technology and family responsibilities.

*‘‘I always thought*, *there are lots of students who just came out of high school*, *and so they’ve still got their study habits*. *I thought maybe they are still in a more academic mindset*. *Whereas I kind of lost mine because I took a break [working]*. *So*, *I think just adjusting your mindset to a very medical way of thinking*. *I found that’s probably the most difficult thing*.*”* [F1, FGD, Med]

However, despite the challenges identified, the students stated that internal motivation and setting goals made the transition process easier.

*‘‘I try not to think too far ahead and try to just make goals*: *‘okay this week’s goal is to just get through the content*, *or this week I might want to concentrate on a few area things’*, *so just having bite-sized chunks because I know if I look too far ahead I’ll get too overwhelmed*.*”* [F1, I, Med]

*Control*: *What aspect of transition could students control*?. Generally, the transition was within the control of the participants as they all made conscious efforts and informed decisions about their chosen fields and institution of study. Nevertheless, some had mixed emotions about coming to university, particularly international students and mature-aged students who had to travel long distance away from family to come to university, and this impacted on ease of adjustment to the transition.

*‘‘I realized that I was leaving my entire life and moving across the world*. *So*, *I wasn’t actually excited to be here until the first week of school when we got to look at the dental clinic and classes started*.” [F1, FGD, Dent]

*Role change*. The transition involved role change for all participants but it was a positive experience for most of the school-leavers who were excited about the newly found “freedom” and “independent lives” as they had to move away from home for the first time.

*‘‘During Year 12*, *I always knew I wanted to leave home*. *So*, *I was always looking forward to it*, *I was never scared*. *I know a lot of my peers were quite homesick but I didn’t feel that*. *I just enjoyed it*.*”* [F3, FGD, Med]

For, the mature-aged students, the role change was massive and it involved some degree of stress as they had to make major life changes to attend university.

*‘‘But it’s a reminder when I think about it*. *I gave up a lot to come here*, *and I think that adds pressure because I sold the home and left a job where I was very comfortable*. *I was making a good income*. *I was very established in my community*.*”* [F3, FGD, Pharm]

*Duration*. The expected duration of the transition affects the ease of assimilation. Assurance of the temporary nature of the change process made it bearable for the participants, especially the international and matured-aged students.

*‘‘So*, *when I realised that I could apply to Bachelor’s Dental degrees in Australia and you could go straight in after high school*, *I was like*: *‘woah*, *here I have to finish four years first but there I could just go in straight away*. *I wish I had known earlier*.*”* [F2 FGD, Dent].

*Previous similar transition experience*. Across the three disciplines, the mature-age students who had obtained previous degrees made comparisons and expressed some anxiety about the new ways of learning and delivery of content material.

*‘‘The mode of learning is different*. *In my previous degree*, *we had a fairly big cohort*. *We didn’t have recorded lectures*, *everything was quite paper-based*. *There were no apps*, *people didn’t bring laptops and iPads and devices into classes*. *There was no Soapbox or Kahoot [online Quiz software]*. *Everything is recorded; I’ve realised that people don’t even come to class*. *Technology is not my strong point*, *but I guess I know enough to be able to navigate my way through*. *And I will ask some of the other wizzes if I need to*: *‘what does this do*? *how do I do that*?*’ So*, *coming into university this time and seeing everyone with their fancy devices*, *I went*: *‘Oh*, *what do I need to learn*?*”* [F1, I, Med].

*Concurrent stress*. Financial burden and health insurance issues were identified by international students as concurrent stress factors during the transition process.

*‘‘Number two*, *finances*. *Yeah*, *it’s expensive for us [laughs]… but yeah*, *you take a loan and you just hope for the best*.*”* [F1, I, Dent];

*‘‘The problem is that the agency didn’t explain to me about Australian health [system]*. *They just told me you need to pay when you go—and I genuinely don’t have any idea of health insurance system here*.*”* [F2, FGD, Med]

*Positive outlook*. The participants had positive attitude towards the transition process and this motivated them to study. For the mature-aged students, returning to the university to study was what they had always wanted, a fulfilment of lifelong dream.

*‘‘Yeah*, *I wanted to get here for 20 years*. *I’m here*, *and I’m staying*.*”* [F4, FGD, Pharm]

*‘‘So those times*, *or really at the end of the day when I’m mulling over the day’s events*, *and you sort of feel really overwhelmed and I can sometimes feel a little bit alone and overwhelmed by ‘Why am I doing this*? *Can I do this*?*’ But the next day you wake up and you just keep doing it*.*”* [F1, I, Med]

#### Self: What does the individual bring to the transition?

*Personal characteristics*. Study participants’ personal characteristics such as origin (international or domestic), gender and age greatly influenced how they perceived the transition process. Some of the international students, particularly those from non-English speaking backgrounds, narrated their initial difficulty in adjusting.

*‘‘There is an English barrier*. *So*, *at the beginning it’s really hard to adjust myself here*. *But am much better now*, *I have improved because of the academic advisor and counselling service here*.” [F2, FGD, Med]

Male students perceived that they were able to easily adjust to academic aspects of university.

*‘‘I didn’t feel like I was in a Pharmacy course*, *I thought I was just repeating high school and just going more in-depth into chemistry and biochemistry*.” [M1, FGD, Pharm]

Female participants on the other hand, were more impacted by the transition and more negative about the experience.

*‘‘Content is harder and I feel like I put more effort in than I did in high school and not getting the result that I’m used to getting was a very big setback*.” [F4, FGD, Med]

*Psychological resources*. Many of the participants identified personal characteristics which they have drawn upon to aid them in withstanding the stress experienced during the transition process. These resources include improved organisational skills, resilience and external commitments/activities.

*‘‘I tried to be organised*, *I do weekly summaries*. *I tried to be on top of it*. *I just had this plan of attack*. *I think having a plan and sticking to your plan is good*. *I feel like I managed to keep it under control*. *By exam time*, *I was ready to take whatever it was because I’ve done all I can*, *so I may as well try my best now*.” [F5, FGD, Med];

*‘‘I am foregoing a salary and if I’m not treating this study like a job*, *if I’m treating it like a party or I’m not being serious about it then I question why I’m here*.*”* [M1, FGD, Dent]

#### Support mechanisms

Students from all disciplines and age groups emphasised the value of support received from family, friends, peers and the institution

*Family support*. Some mature-aged students with families and children stated that they relied on the support provided by members of their families while studying.

*‘‘I have my mother-in-law live with them [kids]*.*”* [F1 I, Dent];

*‘‘I’m appreciative of the fact that I have my Mum supporting me and I don’t have to pay rent*. *I will never starve because she puts food on the table even though I feel guilty*.*”* [F1, I, Med]

School-leavers also acknowledge the support received from their family.

*‘‘I think emotionally it’s been good*, *because I’m living with family up here at the moment*. *I’ve got my uncle*. *So it’s good to come home and see a familiar face*.*”* [M1, FGD, Pharm];

*‘‘I feel less home sick because I am always seeing my brother and sister-in-law and I talk to my parents everyday on the phone*.*”* [F1, FGD, Dent]

*Peer support*. There was mixed feelings about peer support While school-leavers reported having good social interactions with each other, in contrast, the mature-aged students stated that they had limited social interactions with the younger students due to the age difference.

*‘‘Sometimes it’s hard to study everything at once by yourself*, *whereas you’re interacting with friends who say a couple of things*. *Then you’re having fun at the same time*, *and I find that I remember much better at the end of those sessions*.*”* [F3, I, Med]

*‘‘Home group gives you an opportunity to interact with other students and maybe wind down from the week*. *It is a good thing*. *It’s important to have that social interaction*, *it helps with the transition into uni*.*”* [M1, FGD, Dent];

*‘‘I’d just like to say one other thing with the social aspect to kind of contrast what you were saying*. *As a mature-age student coming into a university with a bunch of 17*, *16 and 18 year olds*, *there’s a lot of negative feedback from a lot of the young people that are like*, *hah*, *these old adults that can’t do what we can do now*.*”* [M1, FGD, Med]

*‘‘Home group was an interesting experience*. *I guess it puts you into contact with people that you wouldn’t ever interact with*, *that come from so many different walks of life*.*”* [F1, I, Med]

*Near-peer support*. Despite the varied opinions shared by school-leavers and the mature-aged students about peer support, most of the participants acknowledged the support provided to them by senior student mentors and home group facilitators as key to easing their transition. These near-peer support facilitate both social interactions and academic learning.

*‘‘I think it was more helpful that we got to talk to older years’ students about their experiences and hearing what they got to do in clinic was always super exciting because that’s where I’m going to be at the end of four years*. *It made more sense because you could connect why you were learning the things you were now*.*”* [F1, FGD, Dent];

*‘‘They know what it’s like to be in our position and looking at a 24 page GLS and being really scared*. *So*, *I seek out more help from the older students that I know*.” [F5, FGD, Med]

*Faculty support*. The students indicated that the orientation program organised by the Faculty at the start of year was very helpful to their transitioning.

*‘‘Orientation was helpful*. *We asked a lot of questions*, *they clarified things*. [F3, I, Med];

*The orientation components were valuable*.*”* [F3, FGD, Pharm];

*‘‘I feel the college itself did everything it could*. *I really liked the activities and I got to meet heaps of people*.*”* [F2, I, Med]

However, there were concerns expressed about the insensitivity of some members of staff to the plight of international students from non-English background.

*‘‘I will not say about the staff name but he or she like we hadn’t had many conversations but suddenly [they asked]*, *“What about defer your degree and then go to Townsville private language school for a while to improve your English*?*”*. *So immediately that ruined all my day*.*”* [F2, FGD, Med].

#### Strategies for coping

Students used different strategies to cope with the demands of the transition process and the associated stress factors. Some of the coping strategies were used to *modify* the situation; *control* the meaning of the situation or *manage* reactions to the stress.

*Modify*. Most of the mature-aged students had to modify their learning style

*‘‘The mode of learning is different*. *In my previous degree*, *we had a fairly big cohort*. *We didn’t have recorded lectures*, *everything was quite paper-based*. *There were no apps*, *people didn’t bring laptops and iPads and devices into classes*. *There was no Soapbox or Kahoot [online Quiz software]*. *Everything is recorded; I’ve realised that people don’t even come to class*. *Technology is not my strong point*, *but I guess I know enough to be able to navigate my way through*. *And I will ask some of the other wizzes if I need to*: *‘what does this do; how do I do that*?*’ So*, *coming into university this time and seeing everyone with their devices*, *I went*: *‘Oh*, *what do I need to learn*?*”* [F1, I, Med].

*Control*. Mature-aged students spent time alone to control the challenging situation, while the school-leavers stated that residing in the residential colleges on campus made the *transition easier*.

*‘‘I like to study on my own*, *I don’t like studying in groups or studying at the library*. *The best place for me to study is in my own place*. *That’s why finding my own accommodation has been very important*, *without having that accommodation*, *I would struggle*.*”* [M1, FGD, Dent];

*‘‘I think being at college also probably helped with the transition of moving away from home*, *because it’s kind of like a middle ground*, *like an intermediate between still being on your own but having someone making sure that you’re still alive…”* [F5, FGD, Med];

*‘‘Yeah*, *and I completely agree with that*. *I think college provides with another support system that I’ve appreciated*, *whether it be like notes that have been passed on to me or an older student that I can approach*. *I think the thing that I most appreciated at college was the extracurricular things that come along with it*, *whether it be sport or music or culture or leadership that is one aspect of my life that I would have missed so much if I didn’t go to college*.*”* [F6, FGD Med]

*Manage*. Most of the younger students took time to participate in social activities in order to de-stress.

*‘‘I joined a couple of different faculty clubs and I do volunteer work*, *so I try to meet other people throughout the university*, *so I interact that way*.*”* [M1, FGD, Med]

*‘‘On the days when I have free time*, *I like to go either exercising or just do nothing and not talk to anyone and have no one ask me anything*. *I’m just alone by myself…It’s so beautiful and tranquil*.*”* [F1, FGD, Pharm]

## Discussion

This study explored how students enrolled in their first year of medicine, dentistry, and pharmacy perceived their transition into the university environment, and the factors that affected their transition. Majority of the students were highly motivated and had a strong attachment to attending university. Therefore, there were no thoughts about dropping out of university among this group of students. In addition, the students reported highly perceived adjustments to the academic and social contexts of university life. Generally, highly developed organisational skills, goal setting ability, personal resilience and coping strategies such as engaging in social activities and utilising adaptive study techniques assisted the students in making these adjustments; which in turn produced feelings of greater control over the challenges associated with the transition. In addition, facilitating a change in personal role/lifestyle positively affected the transition process. Nonetheless, international students and mature-aged students felt less emotionally adjusted to university (both physically and psychologically) due to the stressors associated with poorer social integration, differences to previous academic experiences, and highly perceived concurrent financial and family sacrifices compared to school-leaving domestic students [31].

Cronbach alpha values for the SACQ domains were consistent with previous research findings [12], thereby confirming the internal consistency reliability of the instrument. The observed high correlations both within and between the SACQ sub-domains also reflect the degree of alignment between the measured constructs. This is an indication that academic, social, emotional and goal/institutional adjustments are all inter-related and substantially contribute to the transition experiences of students. It also implies that people engage with various contexts of their life in an active way, such that events happening in one context affect the other [32, 33]. It could be interpreted that students’ experiences of academic difficulties could be impacted by their social relationship and emotional stability. Similarly, mastery in one of the domains/subscales is associated with feelings of wellbeing in the others [34, 35]. For example, the high correlation between the psychological and performance sub-domains show that self-belief and motivation translate to successful progression through the course [36]. Hence, the major task for universities is to help students overcome difficulties in all domains of transition. Effective scaffolding of learning and provision of relevant support mechanisms, including self-care programs to tackle unpleasant transition experiences can become opportunities for students to develop new abilities and resources [34–39].

As indicated by Schlossberg et al. [28], an individual’s ability to cope with transition depends on the changing interaction and balance of their assets and liabilities. This study reveals that students’ demographic and psychological characteristics emphasise their positive assets and strengths as well as areas of vulnerability, which subsequently impact on their academic, social, emotional and institutional adjustment levels. For example, male students had higher perceived social adjustment levels in comparison to their female counterparts [14]. Drawing from stress and coping models of development, researchers have reported increased vulnerability to anxiety and stress in females compared to males, particularly when they are faced with uncertainty [40]. Additionally, prior academic experiences with established learning techniques may conflict with newer approaches. Mature-aged and international students can suffer stressors relating to financial stability and significant non-academic commitments [5]. Early identification of limitations could be enhanced through accessible and well- structured institutional support mechanisms [37] which would in turn foster successful transition and aid students in building confidence that prepares them for the journey ahead.

Participants in this study used social support from peers, family and friends to ease their transition experiences. Social support serves as a mechanism to manage diverse aspects of life because it has a positive impact on physical, mental and social wellbeing [41] and is therefore an important correlate of students’ adjustment to university [42]. It refers to the affection, attention and assistance which people utilise in supporting one another [43]. Student support groups help to provide strong academic learning environments inside and outside the classroom [10, 44]. The power of peer and near-peer support within the student cohorts can also serve to facilitate professional identity development [45]. However, it should be noted that even though social support is essential for new experiences and adaption into new environments, not all relationships between individuals facilitate meaningful social support. A relationship is considered as a source of social support only if the receiver perceives that it satisfies his/her needs [46]. Therefore, it is suggested that when educators provide social support resources to students, there is need to ascertain that the resources are fit for purpose. Schlossberg et al. [28] reported that if students feel good about their university experience, it is easier for them to make the transition. A regular evaluation of students’ transition experiences could foster student retention and improve their academic outcomes. The CMD has strong support systems where Academic Advisors are seen as highly accessible and approachable for more than just academic advice, serving to mitigate possible difficulties experienced by students in their first year by providing confidential consult and referral to other support mechanisms in the university, community, and health sector [24]. These support systems may have aided the students in developing effective coping skills. The results of this study emphasise the importance of organising training programs for academic staff/first year coordinators to aid their acute awareness of the personal and psychological factors that could impede smooth transition experiences of first year university students with possible adjustment of the levels of support provided accordingly [45].

### Strengths and limitations

A mixed method approach offers the opportunity to use qualitative study to investigate the underlying reasons for results from the quantitative study. Hence, improving the value of insights derived from these results for educational and research practices. Additionally, the use of the 4 S transition model to interpret the results of the qualitative component of this study has facilitated an in-depth understanding of the major factors that impact on students’ transition into university and how students connect to the help they need to cope with this process. However, the study focuses on health profession students from a single university and there was a disproportionate number of male and female participants, potentially limiting the generalisability of the findings. In addition, self-selection bias might have occurred due to the provision of incentives to participants, which may have distorted or inflated the relationships between variables. However, the emphasis on voluntary participation in the study may have combated this drawback. Furthermore, the study was a cross sectional design, thereby limiting the longitudinal investigation of the patterns of adjustment to university.

## Conclusions

Transition into university is significantly challenging for health profession students, particularly because of their heavy academic workload. Nonetheless, this group of students are highly motivated and therefore do not consider dropping out as an option. Therefore, it is important for health professions faculties to be actively involved in implementing self-care programs that enhance these students’ well-being and mental health. The study findings also substantially extend our understanding of the exacerbated challenges for international, female and mature-aged students who may suffer additional stressors relating to financial stability, uncertainty and significant non-academic commitments. This implies that students’ demographic and psychological characteristics determine their transitional experiences and adjustment to university. It is therefore recommended that tertiary institutions proactively facilitate the provision of tailored support programs that nullify social integration barriers and aid different student groups to interact with one another thereby fostering successful transition and confidence building that prepares them for the journey ahead. Our study findings may guide institutional stakeholders in developing effective academic and social support systems that enhance first year health profession students’ university experience, irrespective of their demographic groups. Future research could focus on longitudinal investigation of the patterns of adjustment to university.

## Supporting information

S1 Checklist COREQ checklist. (DOCX)

S1 Table Summary results for adaptation to university by participants’ characteristics. (XLSX)

## Abbreviations

CMD: College of Medicine and Dentistry
Dent: Dentistry
F: Female
FGD: Focus Group Discussions
HPSC: Health Professional Self-Care
I: Interview
JCU: James Cook University
M: Male
Med: Medicine
Pharm: Pharmacy
SACQ: Student Adaptation to College Questionnaire
